# Regulatory and coding genome regions are enriched for trait associated variants in dairy and beef cattle

**DOI:** 10.1186/1471-2164-15-436

**Published:** 2014-06-06

**Authors:** Lambros Koufariotis, Yi-Ping Phoebe Chen, Sunduimijid Bolormaa, Ben J Hayes

**Affiliations:** Faculty of Science, Technology and Engineering, La Trobe University, Melbourne, Victoria 3086 Australia; Department of Environment and Primary Industries, AgriBio Building, 5 Ring Road, Bundoora, Victoria 3086 Australia; Dairy Futures Co-operative Research Centre, 5 Ring Road, Bundoora, Victoria 3086 Australia

**Keywords:** Variants component analysis, Regulatory genome, GWAS prioritization, Enrichment depletion

## Abstract

**Background:**

In livestock, as in humans, the number of genetic variants that can be tested for association with complex quantitative traits, or used in genomic predictions, is increasing exponentially as whole genome sequencing becomes more common. The power to identify variants associated with traits, particularly those of small effects, could be increased if certain regions of the genome were known *a priori* to be enriched for associations. Here, we investigate whether twelve genomic annotation classes were enriched or depleted for significant associations in genome wide association studies for complex traits in beef and dairy cattle. We also describe a variance component approach to determine the proportion of genetic variance captured by each annotation class.

**Results:**

*P*-values from large GWAS using 700K SNP in both dairy and beef cattle were available for 11 and 10 traits respectively. We found significant enrichment for trait associated variants (SNP significant in the GWAS) in the missense class along with regions 5 kilobases upstream and downstream of coding genes. We found that the non-coding conserved regions (across mammals) were not enriched for trait associated variants. The results from the enrichment or depletion analysis were not in complete agreement with the results from variance component analysis, where the missense and synonymous classes gave the greatest increase in variance explained, while the upstream and downstream classes showed a more modest increase in the variance explained.

**Conclusion:**

Our results indicate that functional annotations could assist in prioritization of variants to a subset more likely to be associated with complex traits; including missense variants, and upstream and downstream regions. The differences in two sets of results (GWAS enrichment depletion versus variance component approaches) might be explained by the fact that the variance component approach has greater power to capture the cumulative effect of mutations of small effect, while the enrichment or depletion approach only captures the variants that are significant in GWAS, which is restricted to a limited number of common variants of moderate effects.

**Electronic supplementary material:**

The online version of this article (doi: 10.1186/1471-2164-15-436) contains supplementary material, which is available to authorized users.

## Background

There is increasing evidence that genetic variation in most complex quantitative traits is the result of many mutations of small effect that individually explain only a small portion of the genetic variance [1–5]. Identification of such mutations has proved challenging in human, livestock and plant genetics [6]. Genome wide associations studies (GWAS) have been successful in detecting some of these mutations for some traits, however the mutations that can be detected with this approach are limited to those that explain enough of the variance to exceed the high significance thresholds necessary to avoid false positives with multiple testing, and are biased towards common variants as a result of ascertainment of single nucleotide polymorphisms (SNP) for widely used arrays [3–8]. A different approach is genomic prediction, where genetic merit of individuals is predicted based on the effect of all the variants estimated simultaneously [7–10].

The number of genetic variants (SNP, insertions, deletions and structural variants) that can be tested for association with traits or used in genomic prediction is growing very rapidly, particularly with the increasing numbers of whole genome sequenced individuals currently available. In humans, the 1000 genomes project consortium has reported 38 million SNPs along with 1.4 million short insertion and deletions [11]. In *Bos taurus* cattle, 28.3 million SNPs, insertions and deletions have been detected, and could be imputed into large data sets for GWAS studies or used for genomic prediction [12]. This presents at least two major challenges; 1) genomic prediction with such a large number of variants is currently not practical due to computing limitations 2) For GWAS, although less computationally demanding (since the analysis is highly parallelizable), very stringent significance thresholds are required to avoid false positives due to such a high level of multiple testing, and as a result many variants with small effects (or more accurately those that explain a small proportion of the variance) would be missed. This also includes rare variants that have large effects but explain a small proportion of the variance [13, 14].

One way to reduce the number of variants for either genomic predictions or GWAS is to use the underlying biological information to annotate the variants into classes and prioritize for testing the functional classes with a higher *a priori* probability of containing trait associated variants (TAV), e.g. those variants significant in a GWAS. In humans, some of the variants that have significant impacts on traits and diseases are found in the protein coding regions of the genome [15], with many studies focusing on the protein coding and conserved annotations to determine variants that are associated with traits [1, 15, 16]. Other similar studies in humans have found that the missense (non-synonymous) variants to be the most enriched annotations for trait associated SNPs [17, 18]. However, a large number of variants with significant associations are found in the non-protein coding regions of the genome. Using regulatory and chromatin structure data, such as the results from the ENCODE project [19], studies have shown that certain genomic annotations such as chromatin structure, DNA methylation, histone modification and other regulatory regions, are enriched for TAVs [18, 20–25]. In livestock however, information on which class of variants that are more likely to be associated with complex traits is almost completely lacking, including for beef and dairy cattle.

In this paper, our aim was to identify regions of the bovine genome that are more likely to contain TAVs. This information could be used for prioritizing variants for future GWAS or for genomic prediction. We annotated variants on the Illumina Bovine HD array (777K SNP) into 17 classes; intergenic (variants that occur in-between genes), intragenic (variants found within genes), variants found 5 kilobases (kb) upstream of a TSS, variants found 5 kilobases downstream of a gene, introns, exons, protein coding sequence (CDS), synonymous variants, missense variants (non-synonymous variants), non-coding conserved variants, microRNA predicted target variants [26], both 5’ and 3’ untranslated regions (UTRs), predicted transcription factor binding site (TFBS) [27], frame-shift, mature microRNAs [28], splice sites and stop sites. We assessed the level of enrichment and depletion of TAVs in twelve classes that contained enough SNPs for analysis, using the results of large GWAS in dairy and beef cattle [29, 30]. To at least partially overcome some of the limitations of GWAS (where the proportion of variance explained must be large to overcome stringent significance thresholds, so variants with smaller effect or rare variants with large effects can be overlooked) we developed two novel variance component approaches to determine if SNPs in any of our functional classes explain more variance than the same number of randomly chosen intergenic SNPs, and to determine which of these annotations capture the greatest proportion of genetic variants on a per SNP basis.

## Results

### Annotation of variants and distribution across the genome

We annotated SNP from 777K Illumina Bovine HD array into 17 classes (Methods). Of the 17 classes, 5 classes; frameshft SNPs, exons, mature microRNA SNPs, all splice sites and all stop sites were not included for further analysis due to having very small numbers of variants or in the case of exons already been represented by the CDS and UTR classes. This leaves us with a total of 12 classes for further analysis (Table 1). The beef data set had a larger number of SNPs than the dairy, because a greater number of SNPs were polymorphic [29, 30]. Intergenic variants (those found in non-coding DNA), were by far the most common (~67% of SNP) as expected [14]. Intragenic variants constituted 32% of the total number of variants. In previous annotation studies using bovine variants it was found that 64.4% of the bovine variants were intergenic and 28.0% of the variants were in intron regions [31], close to our results for the proportion of SNP in intergenic (67.8% and 67.3% for dairy and beef respectively) and introns (30.9% and 31.3% for dairy and beef respectively).

**Table 1:** **Total number of variants annotated in different genomic classes in the beef and dairy data sets**

Class	Number variants in dairy	Number variants in beef
Total Genomic	632003	729254
Intergenic	428255 (0.68)	490982 (0.67)
Intragenic (genic regions)	203534 (0.32)	237914 (0.33)
Upstream variants	23799 (0.04)	27861 (0.04)
Downstream Variants	26453 (0.04)	30780 (0.04)
Introns	195012 (0.31)	227938 (0.31)
Exon	8913 (0.01)	10416 (0.01)
Protein Coding Sequence (CDS)	6364 (0.01)	7490 (0.01)
Synonymous	3968 (0.01)	4768 (0.01)
Missense	2392 (0.004)	2718 (0.004)
UTR (5' & 3')	2386 (0.004)	2748 (0.004)
Non-Coding Conserved	6331 (0.01)	7399 (0.01)
microRNA Predicted Target	1932 (0.003)	2213 (0.003)
Transcription Factor Binding Sites	229 (0.0004)	271 (0.0004)
Frameshift SNPs	1	1
Mature microRNA SNPs	1	2
All Splice Sites	720 (0.001)	855 (0.001)
All Stop sites	86 (0.0001)	88 (0.0001)

The total number of SNP differ between data sets because the beef data included more breeds, so more SNP were polymorphic. Annotations were obtained from Ensembl version 73 [51], with the exception of mature miRNA SNPs and miRNA predicted targets which came from miRBase databases [28, 53], transcription factor binding sites were from Bickhart [27] and the non-coding conserved which were obtain from a phastCons study [55].

### Evidence for enrichment or depletion of functional classes for trait associations

Multi-breed GWAS results were available for 11 dairy traits and 10 beef traits (Table 2). For the beef data a total of 10,181 beef cattle with trait records were genotyped, including *Bos taurus, Bos indicus* (Brahman) and composite animals [29]. For the dairy data, 17,425 Holstein and Jersey cattle were genotyped, with the GWAS results coming from a multi-breed analysis [32]. We used a significance threshold of GWAS *P*-value <0.0001 to define trait associated variants (TAVs) for further analysis since this gave enough power to identify enrichment or depletion while limiting the number of false positives (complete results at all significance thresholds are presented in Additional files 1, 2). We performed permutation testing on each class to determine if the level of enrichment or depletion was significantly above or below that that would be observed by chance, given the number of SNP in the class (see Methods).

**Table 2:** **Dairy and beef trait descriptions including the number of phenotype records for the GWAS analysis**

Trait ID	Animal	Trait name and description	Number of phenotypes
Fat	Dairy	Fat Volume	16812
Fat Percent	Dairy	Fat Percent	16812
Milk	Dairy	Milk Volume	16812
Protein	Dairy	Protein Volume	16812
Protein Percent	Dairy	Protein Percent	16812
Angularity	Dairy	Angularity	6910
BCS	Dairy	Body Conditioning Score	6910
Mammary System	Dairy	Mammary System	6910
Fertility	Dairy	Fertility	15430
Survival Direct	Dairy	Survival Direct	15352
SCC	Dairy	Somatic Cell Count	16297
LLPF	Beef	Peak force measured in Longissimus lumborum muscle (kg)	5358
CIMF	Beef	Percent intramuscular fat measured in Longissimus lumborum muscle	5824
CRIB	Beef	Rib fat at slaughter	5464
SEMA	Beef	Exit scanned eye muscle area	4539
SC12	Beef	Scrotal circumference measured at ages of 12	1112
PNS24	Beef	Percent normal sperm at the age of 24 months (%)	964
PPAI_BB	Beef	Post partum anoestrus interval in BB (days)	629
PPAI_TC	Beef	Post partum anoestrus interval in TC (days)	863
AGECL_BB	Beef	Age at first detected corpus luteum in BB (days)	1007
AGECL_TC	Beef	Age at first detected corpus luteum in TC (days)	1108

**Figure 1:**
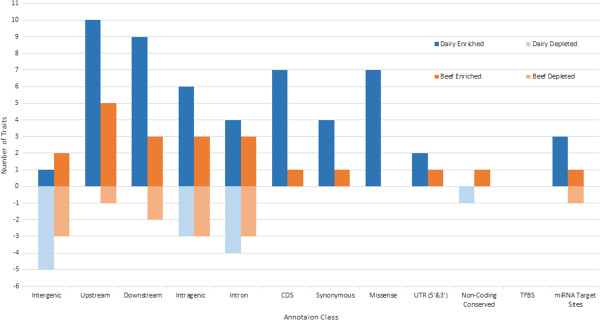
**Enrichment or depletion analysis of trait association variants in annotation classes.** Permutation testing was performed to determine if the number of variants found to be significant in each class was greater than expected by chance for the total number of variants in that class. The number of traits that are significant are shown in blue for dairy and orange for beef. Enriched traits are indicated in the positive dark blue bars for dairy and positive dark orange bars for Beef. Depleted traits are shown below their corresponding class with light blue bars indicating depletion in dairy and light orange bars indicating depletion in beef.

Although the intergenic class had the largest number of variants (67.8% in dairy and 67.3% in beef), this class was significantly depleted for TAVs for majority of traits (Table 3, Figure 1). Although, the dairy trait fertility and beef traits SC12 and PNS24 were the exception, we must note though that the number of animals with phenotypes for these two traits was quite limited (Table 2).The intragenic class (protein coding class) includes the CDS, introns, UTRs (both 5’ and 3’), synonymous and missense classes as a whole. Overall in the intragenic class we find 6 out of 11 dairy traits and 3 out of 10 beef traits to be significantly enriched (Figure 1).

**Table 3:** **Traits that are enriched or depleted for TAVs in both dairy and beef cattle for annotation classes**

Trait	Cattle breed	Intergenic	Upstream	Downstream	Intragenic	Intron	CDS	Synonymous	Missense	UTR (5’ & 3’)	Non-coding conserved	TFBS	micro RNA target
Fat	Dairy	ns	+	+	+	-	+	+	+	ns	ns	ns	ns
Fat Percent	Dairy	-	+	+	+	ns	+	+	+	ns	ns	ns	+
Milk	Dairy	-	+	+	+	+	+	+	+	ns	ns	ns	+
Protein	Dairy	-	+	+	+	+	+	ns	+	ns	-	ns	ns
Protein Percent	Dairy	-	+	+	+	+	+	+	+	+	ns	ns	ns
Angularity	Dairy	-	+	+	+	+	ns	ns	+	ns	ns	ns	ns
BCS	Dairy	ns	+	+	ns	ns	+	ns	+	ns	ns	ns	ns
Mammary System	Dairy	ns	+	+	-	-	+	ns	ns	ns	ns	ns	+
Fertility	Dairy	+	+	ns	-	-	ns	ns	ns	ns	ns	ns	ns
Survival Direct	Dairy	ns	+	+	-	-	ns	ns	ns	+	ns	ns	ns
SCC	Dairy	ns	ns	ns	ns	ns	ns	ns	ns	ns	ns	ns	ns
LLPF	Beef	-	+	+	+	+	+	+	ns	+	ns	ns	+
CIMF	Beef	-	+	ns	+	+	ns	ns	ns	ns	ns	ns	ns
CRIB	Beef	ns	+	ns	ns	ns	ns	ns	ns	ns	ns	ns	ns
SEMA	Beef	ns	ns	ns	-	-	ns	ns	ns	ns	ns	ns	ns
SC12	Beef	+	+	+	-	-	ns	ns	ns	ns	+	ns	ns
PNS24	Beef	+	+	+	-	-	ns	ns	ns	ns	ns	ns	-
AGECL_BB	Beef	ns	ns	-	ns	ns	ns	ns	ns	ns	ns	ns	ns
AGECL_TC	Beef	ns	ns	-	ns	ns	ns	ns	ns	ns	ns	ns	ns
PPAI_BB	Beef	ns	-	ns	ns	ns	ns	ns	ns	ns	ns	ns	ns
PPAI_TC	Beef	-	ns	ns	+	+	ns	ns	ns	ns	ns	ns	ns

Traits were deemed to be significant by permutation testing. The null distribution for the permutation testing was constructed by testing the same number of SNP in each class, but randomly chosen, for the number of significant SNP at *P*<0.0001, and random selection of SNP was performed 1000 times for each class. A class was enriched if the actual number of SNP significant in that class was greater than the number significant in the 950^th^ highest random set, and depleted if the number of significant SNP was less than the number significant than the 50^th^ lowest random set. Traits that are enriched for TAVs in a functional class are indicated with +, those that were depleted are marked with -, and traits where no significance occurred is indicated with *ns*.

The synonymous class was enriched for 4 dairy traits while in beef we see enrichment in only one trait (LLPF). The missense class, however, had one of the highest levels of enrichment after the upstream and downstream classes in dairy with 7 traits to be significantly enriched for TAVs, while in beef we see no enrichment or depletion. Similarly, in the CDS class (the protein coding sequence class which includes both synonymous and missense variants) we find 7 dairy traits to be significantly enriched while for the beef traits we see only the trait LLPF to be enriched. The UTR class (both 3 prime and 5 prime) on the other hand is minimally enrichment for only 2 dairy traits and one beef trait (LLPF).

The intron class was enriched for just as many traits as it was depleted, with 4 dairy and 3 beef traits showing both enrichment and depletion for TAVs. The enrichment for TAVs in this class is perhaps a surprising result given that in human studies intron variants are found to be minimally enriched or depleted [1, 17, 18]. This result, as well as enrichments for the synonymous class, may be due to high linkage disequilibrium between SNPs in these annotations and causal mutations.

Overall, in dairy, the traits fat percent and protein percent were enriched in most of the protein coding annotation classes. The power to detect enrichment for these traits is greatest because they have the highest heritability of all the traits studied here.Of the non-coding regions the upstream and downstream classes were the most significantly enriched, in fact, they were enriched for more dairy traits than the protein coding classes. Similarly for the beef traits we see significant enrichment in the upstream class for most traits, with the downstream class having equal highest level of enrichment to the intragenic and intron classes, (Figure 1). The transcription factor binding site (TFBS) class, however, had no traits significantly enriched or depleted in our analysis, most likely due to the very low number of variants found in this class.

The non-coding conserved class had little enrichment for TAVs. One dairy trait (protein) was actually depleted for TAVs in this class (Table 3), while for beef we find one trait (sc12) to be enriched. The low level of enrichment in this class is surprising given that evolutionary conserved variants have been suggested as among the most important for prioritization [33].

### Logistic regression analysis on GWAS

In the enrichment or depletion analysis above, we examined our annotations independently. However, due to linkage disequilibrium, signals from one annotation may be present in another annotation. A logistic regression analysis was performed to determine the significance and estimated effect that an annotation has on the dairy and beef traits when fitted with all annotation classes together. We selected our significance level at *P*-value <0.0001 for our traits, and converted the data to binary, where if a variant has a GWAS *P* <0.0001 we code it as a “1” otherwise it is coded as a “0” (for not significant). Similarly for our annotation data, if a variant is found in an annotation class we code it as a “1” otherwise if it is not found we code it as a “0”. We recorded the *P*-value along with the regression coefficient from the logistic regression analysis (Methods). Results from this analysis were generally similar to the analysis above, however there were some important differences.

For both dairy and beef traits, our logistic regression analysis shows the upstream and downstream classes to be very significant, with high estimated effects and positive regression coefficients on most traits (Table 4). In particular the dairy traits; fat, fat percent, milk, protein, protein percent and survival direct, all indicating very significant effects in these classes. We also find significance in the CDS and intragenic classes, showing some of the highest estimated effects in this analysis. However, the synonymous and missense classes all show minimal significance, which is somewhat surprising given that in the enrichment or depletion analysis we found the missense class to be significantly enriched for many traits. If the CDS class is removed, the significance and estimated effect for the missense and synonymous classes greatly increase (results of this analysis are shown in Additional file 3).

**Table 4:** **Results from logistic regression analysis**

Traits	Intergenic	Upstream	Downstream	Intragenic	Intron	CDS	Synonymous	Missense	UTR (5'&3')	Non-coding conserved	TFBS	micro RNA target
Fat	0.05 (−0.01)	4.92 (0.15)	3.36 (0.12)	0.55 (0.10)	0.17 (−0.11)	4.70 (0.12)	0.00 (0.02)	0.26 (0.10)	0.27 (−0.21)	0.07 (0.02)	0.22 (0.18)	0.55 (−0.16)
Fat Percent	0.68 (−0.08)	6.44 (0.14)	4.89 (0.11)	12.71 (0.52)	0.89 (−0.46)	11.69 (−0.44)	0.29 (0.36)	0.06 (0.38)	0.67 (−0.40)	0.48 (−0.08)	0.26 (−0.32)	1.53 (0.28)
Milk	0.67 (−0.06)	1.91 (0.03)	2.80 (0.05)	2.16 (0.64)	2.12 (−0.66)	3.28 (0.14)	0.84 (−0.66)	0.37 (−0.54)	0.05 (−0.66)	0.93 (−0.10)	0.09 (−0.09)	1.26 (0.20)
Protein	3.05 (−0.17)	2.60 (0.00)	5.12 (0.01)	4.20 (0.39)	1.41 (−0.49)	2.85 (0.06)	0.51 (−0.47)	0.86 (−0.28)	0.19 (−0.46)	1.11 (−0.11)	0.20 (0.13)	0.48 (0.10)
Protein Percent	0.21 (−0.02)	15.12 (0.21)	9.59 (0.17)	5.10 (0.38)	0.84 (−0.33)	8.60 (−0.10)	0.00 (0.00)	0.46 (0.11)	4.06 (−0.06)	0.57 (−0.07)	0.55 (0.29)	0.84 (0.15)
Angularity	0.89 (−0.25)	2.59 (0.11)	1.31 (0.02)	7.74 (−0.75)	0.78 (0.79)	1.41 (0.86)	0.06 (−0.26)	1.12 (0.37)	0.16 (0.38)	0.93 (0.29)	0.09 (0.21)	0.71 (0.40)
BCS	1.14 (−0.60)	2.16 (0.02)	2.12 (0.02)	0.12 (0.34)	0.24 (−0.88)	3.04 (2.44)	2.18 (−3.07)	0.21 (−2.13)	0.00 (−0.81)	0.73 (−0.94)	1.05 (1.66)	0.14 (0.22)
Mammary System	1.27 (0.31)	2.70 (0.51)	1.16 (0.38)	2.54 (1.01)	0.52 (−0.86)	1.51 (−0.45)	0.00 (0.07)	0.04 (0.04)	1.34 (−0.32)	0.64 (−0.31)	0.24 (−5.29)	1.61 (0.65)
Fertility	2.52 (0.90)	1.46 (0.97)	0.15 (0.59)	2.44 (3.86)	5.45 (−3.37)	0.57 (−2.92)	0.16 (0.31)	0.62 (−0.98)	0.04 (−3.33)	0.27 (0.23)	0.17 (−3.91)	0.67 (−3.93)
Survival Direct	4.19 (0.28)	7.40 (0.41)	6.19 (0.39)	3.21 (0.43)	0.32 (−0.23)	0.75 (−0.71)	0.41 (0.62)	0.11 (0.55)	4.97 (0.22)	0.95 (−0.16)	0.13 (0.14)	0.53 (−0.20)
SCC	0.88 (0.30)	0.04 (0.17)	0.82 (0.37)	0.55 (0.06)	0.06 (0.16)	0.05 (0.09)	0.00 (0.00)	0.06 (0.10)	0.72 (0.55)	2.05 (0.51)	0.23 (−5.00)	0.66 (−0.88)
LLPF	0.51 (0.12)	28.17 (0.88)	16.27 (0.74)	10.89 (0.37)	0.06 (0.11)	3.11 (0.22)	0.05 (0.22)	0.40 (−0.08)	1.66 (0.19)	0.99 (−0.43)	0.07 (0.14)	1.50 (0.59)
CIMF	0.00 (0.01)	3.38 (0.46)	0.1 (−0.05)	8.15 (−0.19)	0.32 (0.57)	0.71 (0.55)	0.00 (0.08)	0.04 (0.03)	0.08 (0.35)	0.1 (−0.10)	2.96 (1.87)	0.31 (0.30)
CRIB	0.18 (−0.1)	1.16 (0.21)	0.07 (−0.13)	0.19 (0.06)	0.04 (−0.13)	0.42 (−0.46)	0.08 (0.78)	0.81 (−0.77)	0.28 (0.17)	0.75 (−0.67)	0.20 (−4.17)	0.08 (−0.16)
SEMA	1.52 (1.55)	0.69 (−0.15)	0.43 (1.24)	2.44 (1.07)	0.04 (−0.27)	0.07 (−1.02)	0.04 (1.21)	0.44 (−1.23)	0.35 (0.55)	0.90 (−2.40)	0.11 (−2.32)	0.41 (0.86)
SC12	0.22 (−0.06)	2.13 (0.12)	2.99 (0.17)	11.18 (0.19)	0.47 (−0.46)	0.17 (−0.39)	0.00 (0.00)	0.06 (0.07)	0.04 (−0.30)	1.45 (0.26)	0.05 (0.09)	0.00 (−0.01)
PNS24	3.58 (0.45)	0.49 (0.40)	0.40 (0.38)	105.99 (0.65)	1.57 (−1.10)	0.04 (−0.48)	0.04 (0.12)	0.27 (−0.06)	0.27 (−0.34)	0.08 (0.01)	1.00 (0.86)	1.27 (−0.74)
AGECL_BB	2.75 (−0.81)	0.30 (−0.78)	0.67 (−0.83)	1.30 (−0.48)	0.11 (−0.22)	0.12 (−0.23)	0.04 (0.12)	0.17 (−0.09)	0.84 (0.29)	0.00 (0.01)	0.25 (−4.97)	0.79 (−0.99)
AGECL_TC	0.04 (−0.06)	0.14 (−0.14)	1.49 (−1.11)	0.86 (−0.03)	0.04 (0.15)	0.05 (−1.29)	0.05 (1.45)	0.61 (−1.78)	0.00 (0.09)	0.20 (−0.33)	0.14 (−3.09)	0.66 (0.88)
PPAI_BB	0.58 (−0.99)	1.10 (−1.85)	0.24 (−1.07)	0.71 (−0.71)	0.00 (−0.10)	0.62 (0.23)	0.00 (0.31)	0.19 (−0.23)	0.57 (−2.78)	0.17 (0.25)	0.14 (−2.88)	0.49 (−2.89)
PPAI_TC	0.77 (−0.56)	0.11 (−0.35)	0.21 (−0.52)	5.11 (−1.46)	0.74 (1.33)	0.36 (−0.53)	0.09 (1.98)	0.91 (−2.18)	1.01 (1.78)	0.20 (−0.26)	0.18 (−3.73)	0.67 (−3.70)

For each cell the first value is -log10 of the *P*-value for the annotation class and trait. The second value (in brackets) is the regression coefficient for annotation class and trait. Enriched annotations have positive effects, depleted annotations have negative effects.

The intron, UTR, non-coding conserved, TFBS and microRNA target classes show very minimal effects and were not significant, with a few exceptions (such as protein percent and survival direct in the UTR class and fertility in the intron class).

### Genetic variance explained by functional classes of SNP

We used a variance component analysis to determine how much variance can be explained by the variants in each class, over and above the same number of randomly chosen intergenic variants. This was achieved by deriving a genomic relationship matrix (GRM) [34] for each class, and then estimating the proportion of total variance explained by the variants using the GRM in a restricted maximum likelihood (REML) analysis.

**Figure 2:**
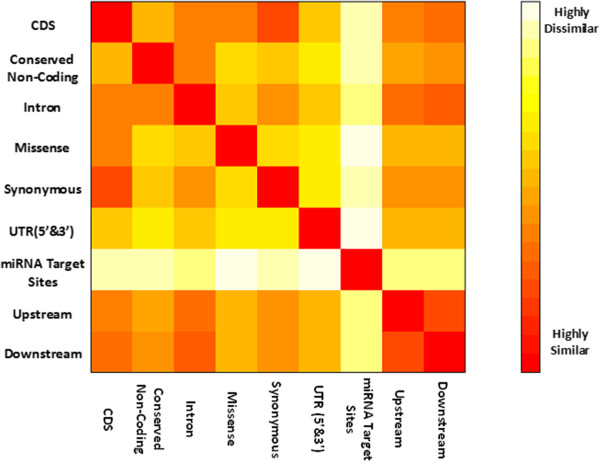
**Heat map visualizing the degree of similarities between the genomic relationship matrices (GRMs) for each annotation.** Highly similar matrices are indicated with a red color and highly dissimilar matrices are indicated with white.

To illustrate the difference, or similarity in information that would be captured by the GRM for each class, we calculated the Euclidean distance between each GRM. We found, not surprisingly, that the genomic relationship matrices between the CDS, missense and the synonymous classes are highly similar since the CDS class consists of both synonymous and missense variants (with 68% of the CDS class consisting of synonymous variants) (Figure 2, Additional file 4). We also observe very similar matrices between the upstream and downstream classes. To determine the significance in the variance explained by each annotation class for our traits, we compared the difference in the variance explained for the traits in each annotation class to the variance explained using the same number but randomly selected intergenic variants. We repeated the random selection permutation test a total of five times to get a standard error (S.E) on the random variance explained to assess if the variants in our classes can explain significantly more variance than the randomly selected variants. This approach was performed for the dairy data set only due to computational restrictions.

**Figure 3:**
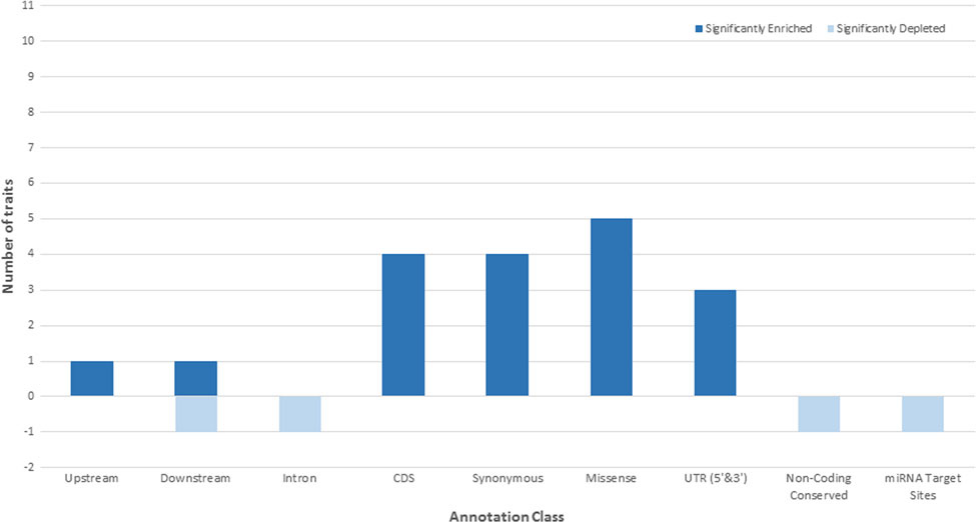
**Traits showing a significant increase or decrease in the variance explained for each of the annotation classes tested, compared with the same number of randomly chosen SNP.** This analysis was replicated 5 times, with significance determine as greater or less than the average of the proportion of variance explained by the randomly chosen SNP +/− 2 times the standard error. Blue bars indicate a significant increase in the variance explained than the same number of randomly chosen intergenic SNPs. Orange bars indicate a depletion in the variance explained for that class.

We find that variants in the protein coding classes explained the most variance, over and above the variance explained by the same number of randomly chosen variants, for most of our traits (Figure 3, Table 5). Interestingly, the number of traits for which this occurs was larger than the number of traits that were significantly enriched for TAVs in this class (Figure 1). For the CDS class, the additional variance explained is very high (8 traits explain more variance and 4 were significant), particularly for the traits fat percent (with a 15.0% difference in the variance explained), milk (4.9% difference) and protein percent (8.5% difference). In the missense class 9 traits explain more of the variance and 5 traits were significant, the highest number of traits in our study and a slight increase from the 7 traits that were enriched for TAVs in our previous analysis (Figure 1). In particular, the trait fat percent explains the largest additional variance in the missense class with an increased difference of 16.9% (Table 5), and has one of the highest heritability in our analysis. Milk volume and protein percent also explained considerably more of the variance (6.8% and 8.3% increase respectively). For the synonymous class the additional variance explained is quite high (7 traits explained more variance with 4 been significant), a similar result to that of our previous number of enriched traits. While in the UTR class (for both 5’ and 3’) we see 5 traits explaining more variance and 3 to be significant, indicating some functional effects on traits and supporting previous studies that the UTRs (particularly the 5’UTR) are significantly associated to traits [1].

**Table 5:** **Difference in variation explained for each trait in each annotation classes tested when compared with the same number of randomly chosen SNP**

Traits	Upstream	Downstream	Intron	CDS	Synonymous	Missense	UTR (5'&3')	Non-coding conserved	Micro RNA target sites
Fat	−0.2	0.0	−2.4	3.8*	5.0*	5.1*	1.8	−1.1	−0.9
Fat Percent	8.7*	4.6*	1.9	18.4*	21.0*	20.0*	5.0*	−3.1*	−3.2*
Milk	2.2	1.2	−1.3	6.4*	7.5*	7.7*	3.4*	−0.3	0.5
Protein	−1.0	−1.4	−3.5*	1.2	1.7	3.2*	2.2	−0.6	0.6
Protein Percent	2.2	1.6	0.2	9.4*	10.9*	9.3*	5.9*	0.2	1.9
Angularity	−1.9	0.3	−1.2	−0.9	−0.5	−0.4	−1.2	0.9	−1.2
BCS	−0.9	−1.3	−1.1	0.6	−0.8	1.4	−1.3	1.2	−0.7
Mammary System	−1.1	−2.4	−0.9	0.1	1.1	−0.1	−0.3	0.1	−1.6
Fertility	−0.8	−0.2	0.5	−0.4	−0.2	0.1	0.0	0.1	−0.3
Survival Direct	−0.8	−1.6	−2.2	−1.9	−0.8	0.1	−1.0	−1.6	−0.7
SCC	−3.0	−3.3*	−2.2	0.5	0.3	0.1	−0.5	−0.1	−0.8

This analysis was replicated 5 times, with significance determined as greater or less than the average of the proportion of variance explained by the same number of randomly selected intergenic SNP across five replicates +/− 2 times the standard error. Values with the symbol “*” indicate that there is a significant increase in variation explained (the differences is greater than 2 times the standard error from the random SNP sets) or a significant decrease in the variance explained (difference is greater than −2 times the standard error from the random SNP sets).

We find that the upstream and downstream classes do not significantly explain more of the variance, which is surprising given the number of enriched traits in the previous analysis. Of all dairy traits, only fat percent was significant in both the upstream and downstream classes, although there was a trend for the other annotations to explain more of the variance when compared to the random intergenic set for the traits fat percent, milk production and protein percent (Table 5, Figure 3). For the non-coding conserved class we see no traits to be significant. What is even more surprising is that the non-coding conserved class explains significantly less variance when compared with the variance explained by the same number of randomly chosen SNP for the traits fat, fat percent, protein, protein percent and milk (Table 5). The trait fat percent, one of the most heritable traits, is significantly depleted in this class, indicating no evidence of these variants to be associated or have any effects on dairy traits. This result, however, is consistent with the enrichment or depletion analysis.We find depletion occurring in one trait and overall no significant extra variance explained for the microRNA target site class, which was not expected given that 3 traits were enriched in the enrichment or depletion analysis (Figure 1). A variance component analysis was not performed on the TFBS class due to the very small number of variants which would not be able to obtain a positive definite genomic relationship matrix.

To extend on the above analysis, we also examined the variance explained per SNP for each annotation class to assess the genetic variance explained by each class in the presence of all the other classes. This assists with eliminating signals from one classes having on another class, and allows us to determine the contributing variance each SNPs have on our traits when categorized in each annotation. We fitted all the GRM simultaneously using GCTA [35], and recorded the heritability (the proportion of genetic variance over the phenotypic variance) and the S.E for each trait across all of our annotations (Methods).

**Figure 4:**
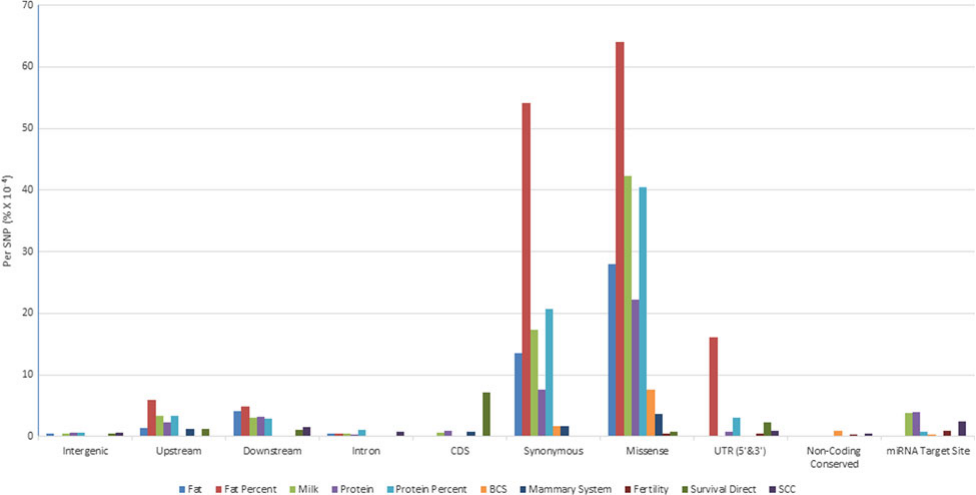
**Proportion of genetic variance explained on a per SNP basis for each of the annotations when fitted jointly in the model.** The genetic variance per SNP is expressed as a% divided by10^−4^. These results show how much variance each SNP contributes to the class for each trait.

Our variance explained per SNP analysis reveals that the missense and synonymous classes explain the greatest proportion of the genetic variance, when the results are expressed as heritability per SNP, compared to all of our classes combined (Figure 4, Additional file 5), particularly for the traits fat, fat percent, milk, protein and protein percent.

We find that the upstream and downstream classes do not explain as much of the variance per SNP as the missense and synonymous classes do, however they do capture more genetic variance per SNP than the intergenic, intron, UTR, non-coding conserved and miRNA target site classes. The UTR (for both 5’ & 3’) annotations surprisingly capture a much smaller portion of the genetic variance per SNP, except for the trait fat percent.The intron, intergenic and non-coding conserved annotations explain the lowest proportion of the genetic variance per SNP while the miRNA target site annotations capture more of genetic variance per SNP than the CDS, intron, intergenic and non-coding conserved classes (Figure 4). These results are almost in complete agreement with our variance component analysis results when the GRM is fitted individually (Figure 3). However, we do find that the CDS class is not in complete agreement with our previous variance component analysis results and this can be explained since this class is represented by the missense and synonymous classes (we see the similarities between the GRMs in Figure 3), thus when fitted simultaneously the effects are already captured.

## Discussion

We tested whether 12 genome annotation classes contained variants affecting complex traits in dairy and beef cattle, using a trait associated variant enrichment or depletion analysis and two variance component approaches. In the TAV enrichment or depletion analysis we observed that the upstream and downstream classes were significantly enriched for most dairy traits and many beef traits, while the protein coding classes are also enriched in dairy, but for fewer traits. In the first variance component approach, where the proportion of variance captured by the SNP in that class was compared to the proportion of variance captured by the same number of random SNP, the protein coding classes (particularly the missense, synonymous and CDS classes) were significant, while the upstream and downstream classes were not for most traits. The second variance component approach, where the GRM for all annotation classes were fitted at once, demonstrated that the missense class explained the most variance on a per SNP basis.

The main difference in results between the TAV enrichment or depletion analysis and the variance component approaches was that the upstream and downstream classes are highly significant in the first approach, but not in the variance component approach across most traits. This difference may arise for two possible reasons. The first reason arises because the actual causal mutations are unlikely to be in our data set. In the upstream and downstream classes, there are a very large number of SNP, so that the chance of at least one of them being in high linkage disequilibrium with a causal mutation (a TAV) is higher than for classes with a lower number of SNP. Secondly, in the TAV enrichment or depletion analysis, a significance threshold is set, while in the variance component approach there is no such threshold. By setting a significance threshold, only variants in linkage disequilibrium (LD) with undiscovered mutations of moderate effect size are effectively considered in the enrichment or depletion analysis. However, given the typical genetic architecture of complex traits, there are likely to be very few of these [36]. In contrast, the variance component approach can capture the cumulative effects of many variants that have smaller effects [34].

Protein coding regions are known to harbor variants affecting complex traits and by focusing on these regions, particularly exons, offers some promise for identifying the genetic variants that are associated with complex traits [37]. The protein coding classes in our analysis proved to explain the most variance in dairy traits for a given number of SNPs and on a per SNP basis (Figure 3 and Figure 4). Missense variants, in particular, are more likely to have some of the most significant effects on traits since these variants alter both the genetic sequence and the amino acid sequence of a protein, having the potential to be deleterious or beneficial. Research in human genetics found that the missense variants are often significantly enriched for trait associations [13, 17, 18]. The variance component analysis indicates that for most dairy traits the missense class both explains significantly more variance than the same number of randomly chosen intergenic variants (Figure 3) and per SNP explains the most variance (Figure 4). In our enrichment or depletion analysis this class was also significantly enriched for most traits (Figure 1). These results clearly suggest the importance of this class for prioritization.

One major limitation of our study is that the variants we investigated are a set of SNPs selected for high minor allele frequency (MAF) and are evenly spaced across the genome (they are SNPs on the Illumina Bovine HD array), rather than the complete set of sequence variants in the population. This can lead to some annotated variants to be in high linkage disequilibrium with variants that can be causative. For example a synonymous variant (a class enriched for some traits) that is associated with a trait is unlikely to be the causative mutation but is more likely to be in high linkage disequilibrium with a nearby causative mutation. This limitation has been reported in many studies [13, 17] and is a possibility for why we see enrichment for TAVs in the intron and synonymous classes, and why we find the synonymous class to explain a large proportion of the variance.

This limitation is most prominent in the intron class (which is enrichment for some traits), where a very large number of variants are annotated for this class, increasing the chance of these variants to be in LD with causative mutations that might not be in introns, particularly for variants near the intron/exon boundaries. However in the variance component analysis, this class explains less of the genetic variance when compared to the same number of randomly selected intergenic SNPs. As mentioned earlier, this class has a large enough number of variants where most of the heritability is captured and it would be difficult to capture any more or less of the variance using this heritability alone when compared to an annotation class with a smaller number of SNPs. Fitting the intron class simultaneously in the model with all other annotations to examine the proportion of variance explained on a per SNP basis confirms that the intron class explains very little variance (Figure 4). In other studies, while the number of variants found in introns constitute the majority of the intragenic variants, they have been shown to exhibit minimal enrichment for associated variants [1] or no evidence of enrichment or depletion [17]. Intron variants with effects on complex traits cannot be completely ruled out however, as elements within introns have in some cases been demonstrated to have regulatory functions [38] and variants found within introns have been shown to affect phenotypes in mammals [39]. A study in schizophrenia find that introns can contribute to the etiology of the disease [40].

The UTRs are implicated to have regulatory functions in controlling gene expression and are known to harbor binding sites for microRNAs [41]. Studies in human genetics have found that the UTR regions to be some of the most enriched for trait associated SNPs [14], particularly the 5’ UTRs [1], other studies on the other hand found that the UTR variants to be modestly enriched and even depleted when using a logistic regression analysis [18]. Our study does indicate similar results, with modest enrichment of the UTR class for TAVs, although, in the variance component approach we find that this class is significant, but for fewer traits than other protein coding classes (Table 5). On a per SNP basis they explain a much smaller proportion of the variance (Figure 4).

The microRNAs (miRNAs) are known to have important regulatory functions with claims that about a third of mammalian genes are actually regulated by miRNAs [42]. In our study, we examined variations in the miRNA coding regions and the miRNA target sites. Very few SNP were found in the miRNA coding regions, (1 in dairy and 2 in beef, Table 1) which could be indicative of their highly conserved sequence nature [43], thus their effects could not be examined. However, we expected to find significant trait associated variants in their predicted target sites. Our results show minimal enrichment for TAVs in miRNA target sites, with our variance component analysis showing no extra variance to be explained by this class. This is surprising given the promising and important regulatory functions that the miRNAs have on gene regulation. Our analysis, however, is limited by the fact that miRNA target sites at present are very difficult to predict, mainly due to the very limited sequence binding of miRNAs to the genome and most tools focusing on 3’UTR regions over full genome scale targets [26]. Similar studies in human genomics have examined miRNA target sites in 3’UTRs only to find no significant enrichment [17].

The upstream and downstream classes are significantly enriched for TAVs for most beef and dairy traits in our analysis (Figure 1), suggesting possible regulatory element mutations with effects on our traits. However, many variants in these classes may be artifacts (SNPs that are not within regulatory regions or other functional regions). These artifact SNPs can have implications in our variance component analysis where significant effects will be averaged out over a large number of (artifact) SNPs that have no effect, resulting in these classes capturing a smaller proportion of the variance. Removal of the artifacts in the upstream and downstream classes would be possible if prior knowledge of where regulatory functions such as promoter, enhancer or TFBS are located on the genome, eliminating the need to have poorly defined annotation classes and instead classes that have potential regulatory functional roles. Further, the GRMs for both the upstream and downstream classes are very similar (Figure 2, Additional file 4), indicating that variants annotated in the upstream class are potentially annotated in the downstream class, further emphasizing the need to have accurately defined annotations in these regions. This is a major limitation in our study involving the non-coding genome, as the current map of regulatory elements in the bovine genome is very limited [27], with almost lacking information for locations of enhancers, promoters and other non-coding regulatory factors. Our results clearly suggest the importance of including variants found in the non-coding genome and that prioritization should not just be limited to variants found within protein coding regions.

Studies in human genetics are beginning to uncover the importance that the regulatory genome has on traits and how variants found in the non-coding regions are as significant as those found within genes. The regulatory genome is highly complex with many interactions contributing to the expression and regulation of genes. Large scale projects such as the ENCODE (Encyclopedia of DNA elements) project [19] are beginning to uncover these complexities having assigned biochemical functions to about 80% of the human genome [15].

Related research found that GWAS SNPs are significantly enriched for promoter annotated variants [17] while enhancer annotations also display strong enrichment signals [18, 44]. Epigenetic studies in humans found that changes in DNA methylation at CpG dinucleotide are heritable and contribute to gene expression [45]. While regions of the genome associated with histone modification patterns have been shown to influence gene expression, and to be enriched for TAVs [22, 24]. Further it has been shown that non-coding variants concentrated around regions of the genome marked by DNase I hypersensitive sites (DHS) are enriched in traits [46]. An ENCODE style analysis of the bovine genome would be extremely valuable for obtaining a greater understanding of the complexities in the non-coding genome that can impact traits through regulation, while providing much greater detailed annotations in the bovine genome that can be used for prioritization.

Variants in the genome for which the sequence is conserved across species have been proposed as an important annotation class for prioritization and are potentially causative [33, 47]. The majority of the conserved variants are actually found in non-coding regions, and it is believed that at least some of these are *cis* regulators for genes [47]. Our study in fact found minimal enrichment in beef and depletion for TAVs in dairy for the non-coding conserved class. Similar studies in humans have also found limited evidence for enrichment of associated SNPs in non-coding conserved regions [18]. Studies are also questioning the importance that non-coding conserved variants have in regulatory functions, such as gene expression, claiming that we cannot use non-coding conserved sequences alone to indirectly predict regulatory functions [48]. It has been shown that removal of ultra-conserved non-coding elements have no significant effects on traits in mice [49]. We have provided some evidence that variants in non-coding conserved regions do not contribute greatly to standing variation for complex traits in cattle.

As previously mentioned, one of the major limitations in our study, is ascertainment bias and the selection of SNPs with high minor allele frequencies that are evenly spaced across the genome resulting in many variants to be in high LD with a nearby causative variant, complicating the interpretation of the analysis. The best way to eliminate this high level of LD in our data and greatly improve this study would be to use whole genome sequence data [50]. Whole genome sequence (WGS) data is not limited to only common SNPs but allows for the detection of most variations found in the genome, including insertions, deletions, structural variants, and rare variants that could potentially have significant effects.

## Conclusion

Our findings suggest annotation of variants based on biological function can assist in prioritization of variants more likely to be associated with complex traits in dairy and beef cattle. The variance component approach indicates protein coding regions explain significantly more variation than a similar number of randomly chosen SNP across many traits. We also found significant enrichment of TAVs in upstream and downstream classes suggesting that these classes can have potentially important regulatory functions. The non-coding conserved regions were, in some cases, actually depleted for TAVs, leading us to assume that these variants are not highly significant in our traits. On a per SNP basis, missense variants explained the greatest variation for many traits. Finally, while protein coding regions are highly important in predicting complex traits, the importance of regulatory function cannot be over emphasized and having reliable regulatory information for further studies in bovine genomics is paramount.

## Methods

### Annotation of variants using Ensembl databases

We annotated dairy and beef SNPs from the 777K Illumina Bovine HD array into; intergenic, intragenic, exons, CDS, UTRs (both 5’ & 3’), 5 kb upstream of TSS, 5 kb downstream of genes, missense, synonymous, frame-shift variants, splice sites and stop codon classes by querying the Ensembl variant database version 73 [51].

### Annotation of microRNAs and microRNA target sites

We used the miRBase database [28, 52, 53] to annotate the microRNA coding variants and for annotation of the target sites we used the MicroCosm target site database [26, 28].

### Annotation of non-coding conserved variants

Non-coding conserved variants were obtained through phastCons [54] for conversed variants across mammalian genomes [55]. We used a threshold conservation score of 0.800 to filter and keep the most conserved variants only.

### GWAS Data and *P*-Value enrichment or depletion for TAVs analysis

*P*-values for dairy traits were from a multi-breed GWAS including 17,425 bulls and cows, of either the Holstein or Jersey breeds [32]. *P*-values for beef traits were from a GWAS of *Bos indicus, Bos taurus* and composite animals [29]. For each trait, we grouped the annotated variants into 7 different thresholds based on the GWAS *P*-value; < 0.1, < 0.01, < 0.001, < 0.0001, < 0.00001, < 0.000001, < 0.0000001 and < 0.00000001 and calculated false discovery rates (FDR) for all thresholds to estimate the number of false positives. The FDR was calculated with the following formula:

Where the *p-value* is one of the *P*-value thresholds (<0.1, <0.01, <0.001, <0.0001, <0.00001, <0.000001, <0.0000001 and <0.00000001), *m* is the number of significant variants found in the each threshold and *n* is the total number of variants in the class. We chose a GWAS threshold of *P* < 0.0001 for our analysis since we found most enrichment or depletion at that *P*-value while FDRs were low, and there were enough significant SNP for analysis in most classes (Additional files 1 and 2).

### Permutation testing to determine enrichment or depletion of TAVs for annotation classes

We performed a permutation test for each trait to determine if the observed proportion of significant SNPs in each annotation was greater than expected by chance alone. This was done by randomly selecting *n* variants (the number of variants found in each annotation class, Table 1) from the genomic data. As an example, the exon protein coding sequence (CDS) class has 6364 variants, therefore we would randomly select 6364 variants from the genomic total (632003 for dairy and 729254 for beef). We selected the total number of random variants with a GWAS *P-*value <0.0001 and recorded that number. We performed this whole process a total of 1000 times to obtain a null distribution of *m* number of variants in each annotation. The *P* < 0.05 value for enrichment was the 950^th^ highest number in the random permutation test, and *P* < 0.05 value for depletion was the 50^th^ lowest number in the random permutation test.

### Logistic regression analysis on GWAS data

Logistic regression was applied to model annotations as variables that influenced trait-association status of the SNPs thus to eliminate signals from one class been present in another. The trait-association status was modelled as a binary variable where if a SNP has a GWAS *P <*0.0001 we consider it significant and coded as “1” otherwise it is not considered significant and coded as “0”. Similarly, we converted all of our functional annotations to binary based on if a variant is found in at a particular annotation class or not. If a variant is found in a particular annotation class we coded it with a “1”, if it is not found in that particular class we code it with a “0”. All annotations were fitted simultaneously using ASREML [56]. The estimated effect (regression coefficient) along with the *P*-value (multiplied by –Log10) were given. An annotation with a low *P*-value and a high estimated effect is considered as enriched for that trait, while an annotation negative estimated effects is considered depleted for that trait.

### Variance component analysis

The purpose of the variance component analysis was to determine if SNP in an annotation classes explain more of the variance than the same number of randomly chosen intergenic SNP. We calculated the genomic relationship matrix (GRM) for each class according to Yang *et al.*[34], using the genotype information for each SNP.

We determined the measure of similarities between the GRMs belonging to the annotations, to see if variants found in one class are also present in another. We did this by calculating the Euclidean distance between each of the annotation GRMs using the following formula:

Where *m* and *p* are the corresponding GRMs for each functional class.

To calculate the proportion of variance explained for each independent annotation class we performed a REML analysis by fitting the following model to the data:

Where y is a vector of phenotypic records, b is a vector of fixed effects including the breed and sex, X is a design matrix allocating records to fixed effects, Z is a design matrix allocating records to breeding values, and g is a vector of random breeding values, , where g is the genomic relationship matrix described above, and  is the genetic variance from each class. This was estimated using ASREML [56], and the estimated proportion of phenotypic variance (heritability) was calculated.

To determine the proportion of variance explained by the same number of random SNP we selected the same number of *n* SNP but from the intergenic variant set. Similar to what was done above, we calculated the GRM for the random intergenic data sets and used ASREML to determine the heritability. This random sampling was run a total of 5 times to get a standard error and we calculated the average of these 5 runs to obtain the random set heritability.

Some classes that have a large enough number of variants, can capture most of the heritability for each trait. This can be a limitation since it would be difficult to determine the true additional variance explained compared to that of the randomly selected intergenic set (which itself can capture most of the heritability with a large enough number of variants (Additional file 6)). Annotation classes with a smaller set of variants are not impacted by this, however classes such as the intron class can be. To get around this limitation we determine the proportion of genetic variance an annotation explains on a per SNP basis. This allows us to observe how much of the variance each SNP contributes to that class and by fitting our annotations simultaneously on the model when in the presence of all other annotations. We used Plink [57] to prepare our data for the software tool GCTA [35] which was then used to calculate the GRMs for each annotation, and to performe a REML (restricted maximum likelihood) analysis.

We fitted our GRMs simultaneously on our model using GCTA, and recorded the ratio of genetic variance to phenotypic variance (heritability) for each trait along with the standard error (S.E). To determine the genetic variance explained on a per SNP basis we used the following formula for each class:

Where *h*^*2*^ is the heritability and *n* is the total number of SNPs in the annotation class. We multiplied this result by 100 to get a percent (%) of the genetic variance explained and divided this results by 10^−4^ for visualization of the data.

### Availability of supporting data

Dairy and beef cattle SNP annotations for the 777K Illumina bovine HD array are provided in the repository hosted by LabArchives, LLC (http://www.labarchives.com/) with DOI: http://dx.doi.org/10.6070/H4N58J9R. Files are stored in .xlsx format.

Dairy GWAS data is published and available in the manuscript by Raven *et al.*[32]. Beef GWAS data is published and available in the manuscript by Bolormaa et *al.*[29].

## Electronic supplementary material

Additional file 1: **Dairy cattle enrichment or depletion for trait associated variants.** Results from the enrichment or depletion analysis across all *P*-Value thresholds tested from the dairy GWAS. Enriched dairy traits are labeled as “+”. Depleted dairy traits are labeled as “-“. Non-significant dairy traits are labeled as “*ns”*. Table is attached in. xlsx format. (XLSX 17 KB)

Additional file 2: **Beef cattle enrichment or depletion for trait associated variants.** Results from the enrichment or depletion analysis across all *P*-Value thresholds tested from the beef GWAS. Enriched beef traits are labeled as “+”. Depleted beef traits are labeled as “-“. Non-significant beef traits are labeled as “*ns”*. Table is attached in. xlsx format. (XLSX 17 KB)

Additional file 3: **Logistic regression analysis on dairy and beef traits with the CDS class eliminated.** We performed a logistic regression analysis on both beef and dairy traits without the CDS class to determine the impact this has on the missense and synonymous classes. The CDS class consist of both missense and synonymous variants, thus signals form these classes are already represented in the CDS class. By eliminating the CDS class in our logistic regression analysis, we clearly see an increase in the significance and the estimated effect for both classes. Table is attached in. xlsx format. (XLSX 12 KB)

Additional file 4: **Matrix of similarities between the genomic relationship matrices.** The matrix shows the level of similarities between the genomic relationship matrices for each function class by calculating the Euclidean distance (measure) between each annotation GRM. The more similar a GRM is to another the lower the Euclidean distance measure is. (XLSX 12 KB)

Additional file 5: **Genetic variance explained per SNP in each annotation class for dairy traits.** The following table shows the heritability for each trait (as a percentage (%)) in each annotation class along with the average genetic variance explained per SNP (calculated as the percentage (%)/10^−4^). Classes that have higher values capture more of the genetic variance that contributes to the heritability. Lower values indicate that they only contribute a much smaller amount to the heritability for that class. Table is attached in .xlsx format. (XLSX 14 KB)

Additional file 6: **Variance component analysis using ASREML in dairy traits.** The following tables show the class heritability for each trait along with the permutated heritability from the same number but randomly chosen SNPs (which was replicated 5 times and significance is determined as greater or less than the average of the proportion of variance explained by the randomly chosen SNP +/− 2*standard error). The heritability percent difference is simply the difference between the class heritability and the permutated heritability. S.E is an abbreviation for standard error. Table is attached in .xlsx format. (XLSX 15 KB)

## Abbreviations

SNP: Single nucleotide polymorphism
TAV: Trait associated variant
GWAS: Genome wide association study
CDS: Protein coding sequence
UTR: Untranslated region
FDR: False discovery rate
TFBS: Transcription factor binding site
GRM: Genomic relationship matrix
REML: Restricted maximum likelihood
MAF: Minor allele frequency
DHS: DNase I hypersensitive sites
WGS: Whole genome sequence
REML: Restricted maximum likelihood
S.E: Standard error.
